# A Survey of Spoofer Detection Techniques via Radio Frequency Fingerprinting with Focus on the GNSS Pre-Correlation Sampled Data

**DOI:** 10.3390/s21093012

**Published:** 2021-04-25

**Authors:** Wenbo Wang, Ignacio Aguilar Sanchez, Gianluca Caparra, Andy McKeown, Tim Whitworth, Elena Simona Lohan

**Affiliations:** 1 Electrical Engineering Unit, Faculty of Information Technology and Communication Sciences, Tampere University, 33720 Tampere, Finland; elena-simona.lohan@tuni.fi; 2 European Space Agency, European Space Research and Technology Centre, 2201 AZ Noordwijk, The Netherlands; Ignacio.Aguilar.Sanchez@esa.int (I.A.S.); Gianluca.Caparra@esa.int (G.C.); 3 GMV-NSL, Nottingham, NG7 2TU, UK; andy.mckeown@gmvnsl.com (A.M.); tim.whitworth@gmvnsl.com (T.W.)

**Keywords:** global navigation satellite systems (GNSS), spoofing, radio frequency fingerprinting (RFF), I/Q (pre-correlation) data, support vector machines (SVM), classifiers, feature extractors

## Abstract

Radio frequency fingerprinting (RFF) methods are becoming more and more popular in the context of identifying genuine transmitters and distinguishing them from malicious or non-authorized transmitters, such as spoofers and jammers. RFF approaches have been studied to a moderate-to-great extent in the context of non-GNSS transmitters, such as WiFi, IoT, or cellular transmitters, but they have not yet been addressed much in the context of GNSS transmitters. In addition, the few RFF-related works in GNSS context are based on post-correlation or navigation data and no author has yet addressed the RFF problem in GNSS with pre-correlation data. Moreover, RFF methods in any of the three domains (pre-correlation, post-correlation, or navigation) are still hard to be found in the context of GNSS. The goal of this paper was two-fold: first, to provide a comprehensive survey of the RFF methods applicable in the GNSS context; and secondly, to propose a novel RFF methodology for spoofing detection, with a focus on GNSS pre-correlation data, but also applicable in a wider context. In order to support our proposed methodology, we qualitatively investigated the capability of different methods to be used in the context of pre-correlation sampled GNSS data, and we present a simulation-based example, under ideal noise conditions, of how the feature down selection can be done. We are also pointing out which of the transmitter features are likely to play the biggest roles in the RFF in GNSS, and which features are likely to fail in helping RFF-based spoofing detection.

## 1. State-of-The-Art-Review and Paper Contributions

The radio frequency fingerprinting (RFF) concept refers to the process of identifying the hardware (HW) characteristic and HW-specific features or signatures embedded in the radio frequency (RF) waves transmitted over a wireless channel [1,2,3,4]. In a strict sense, RFF refers only to the transmitter-specific HW features. In a broader sense, the RFF process has also been studied in the context of channel characteristics or features, typically in the context of indoor positioning [5,6,7,8], as well as in the context of joint transmitter–receiver identification [9]. In this paper, we adopted the first definition of RFF, namely that the ‘features’ to be identified refer to HW specifics of a wireless transmitter. As a side note, this RFF concept is also encountered in the research literature under the names of *specific emitter identification (SEI)* or *physical layer identification*. The purpose of any RFF technique is to identify genuine transmitters (or transceivers) and distinguish them from malicious ones. For example, the authors in [10] performed a thorough analysis of GPS signals using a 30 m dish antenna, illustrating the evolution of the signal quality among the different GPS satellite generations. The paper indirectly showed that with a sufficiently high gain antenna, if the signal-to-noise ratio (SNR) is sufficiently improved, it is possible to identify the specific GNSS signal transmitter.

Especially in the context of global navigation satellite systems (GNSS), intentional interference such as jamming and spoofing has been on the rise in recent years and can have significant adverse effects on the navigation performance of GNSS receivers, as discussed for example in [11,12,13,14,15].

Future aviation applications, and in particular unmanned aerial vehicles (UAVs), will increasingly rely on GNSS-based navigation and positioning solutions [14,15]. Safety-critical applications, such as those from the aviation domain, require a high capability of anti-spoofing and anti-jamming detection, or, in other words, a high identification accuracy of genuine and malicious transmitters.

There are many authentication and anti-spoofing methods in GNSS which are not based on RFF and such methods that have been widely studied in post-correlation, and especially at navigation levels [11,16,17,18,19]. Recently, with the advent of RFF concepts in many non-GNSS wireless communications and with increased capabilities of machine learning (ML) approaches, the RFF solutions have also started to be considered in the GNSS field; in particular, the research problem of whether RFF could work with raw GNSS data, in the pre-correlation domain, before acquisition and tracking, remains an unsolved problem. It is the purpose of this paper to shed more light on whether RFF on pre-correlation GNSS data can work and which are the challenges and limitations in this field. In order to address this research gap of how to apply the well-known radio frequency fingerprinting and ML methods (to date widely used in other research fields) in the context of GNSS receivers, we present here a comprehensive survey of RFF and ML methods, discuss their applicability in the GNSS context, and we introduce a novel methodology to deal with RFF in GNSS, by presenting equivalent block diagrams of the genuine and non-genuine GNSS transmitters. We also give an initial glimpse of what kind of transmitter features are the most important in the context of GNSS transmitters, based on an in-house-made simulator, with Matlab and Python modules. We further summarize the remaining challenges when dealing with realistic environments and point out a few possible paths for future research in this challenging field.

**Figure 1:**
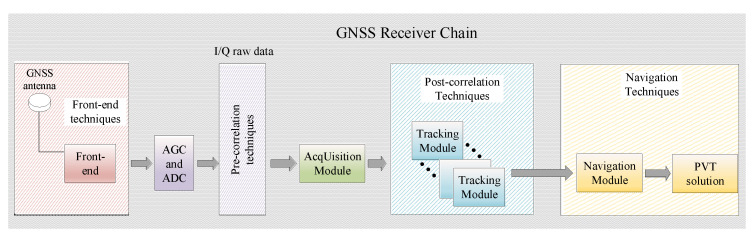
The three domains of a typical GNSS receiver: pre-correlation; post-correlation; and navigation domains.

A schematic block diagram of the three domains (pre-correlation, post-correlation, and navigation) of a typical GNSS receiver is shown in Figure 1. The pre-correlation domain refers to the data at the output of the Automatic Gain Converter (AGC) and Analog-to-Digital Converter (ADC) shown in Figure 1, in other words, to the raw I/Q samples before the acquisition stage of the GNSS receiver. These samples are typically received at a very low signal-to-noise-ratio, but they can carry important information about the ‘features’ of the transmitter, as they are not yet smoothed or filtered with the correlation filters.

A good survey of anti-spoofing methods based on the post-correlation and navigation data in GNSS can be found for example in [19]. However, no pre-correlation methods and no RFF methods were addressed in there. Others surveys of anti-spoofing methods can be found for example in our previous work in [11,20], where again only the post-correlation and navigation anti-spoofing solutions were addressed. Feature-selection methods for RFF based on the navigation domain of a GNSS signal have also been addressed in [21]. Surveys on the RFF methods are more difficult to find in the current literature, and they are typically focused on non-GNSS signals, such as cellular, Internet of Things (IoT), or WiFi signals [22,23,24,25,26].

As seen in the discussions above, there is still a lack of surveys of RFF methods for GNSS transmitter authentication in the current literature, particularly on surveys of GNSS authentication relying on pre-correlation signals. In this paper, we are addressing this lack, via a comprehensive study of the literature in the past two decades, as well as via theoretical insights and the preliminary analysis of algorithms. Our contributions are as follows:

1. Offering a thorough survey of RFF methods applied with GNSS and non-GNSS wireless data in the literature, and discussing which of these RFF methods have potential in GNSS, and in particular in GNSS with pre-correlation data. Finding good anti-spoofing methods based on pre-correlation GNSS data could have tremendous benefits for the future GNSS receivers, by being able to detect and remove non-genuine signals even before processing them further in the acquisition and tracking loops. Our survey is unique in the current literature, as the RFF methods for GNSS have to date not been widely investigated and there is a current lack of unified surveys on this;
2. Proposing a step-by-step problem definition of RFF in the context of GNSS signals, by delving in depth in the sources of possible transmitter hardware impairments, and also discussing the possible channel and receiver–hardware impairments; this problem decomposition into feature-by-feature investigation is also lacking from the current GNSS literature, to the best of our knowledge;
3. Proposing a four-step generic RFF approach, consisting of: feature identification, feature extraction, data pre-processing, and data classification. Classical ML and transforms methods are used in this four-step methodology, but the four-step block diagram is rather novel;
4. Presenting the mathematical models of different GNSS transmitter features, with a particular emphasis of five main identified features, namely: the power amplifier non-linearities, the digital-to-analog converters’ non-linearities, the phase noises of the local oscillators, the I/Q imbalances, and the band-pass filtering at the edge of the transmitter front-end; unified mathematical methods of the transmitter HW impairments are not found in the current literature to the best of the authors’ knowledge;
5. Providing the equivalent transmitter block diagrams for GNSS and spoofers by incorporating the aforementioned five hardware effects into the models;
6. Presenting an illustrative simulation-based analysis based under ideal conditions in order to emphasize the impact of each HW feature on the RFF performance. Three feature extractors to identify the transmitter HW impairments were used, namely the kurtosis, the Teager–Kaiser energy operator (TKEO), and the spectrogram. The classification accuracies given as examples are based on support vector machines (SVM). Such a simplified analysis allows us to identify the strongest features among the five considered ones and to point out the remaining challenges to overcome to achieve the feasibility of RFF methods under more realistic GNSS scenarios;
7. Bringing in a qualitative discussion on the existing algorithms and providing a roadmap towards further research on RFF in GNSS for interference detection and classification.

The rest of this paper is organized as follows: Section 2 presents the use case of a spoofing attack on an on-board GNSS receiver and describes the various spoofing types and anti-spoofing approaches existing in the literature. It also clarifies the fact that the focus of our paper is on pre-correlation approaches using the I/Q sample-level data as inputs, but the proposed methodology and the identified feature extractors and classifiers can also be applied in a broader sense, with post-correlation and navigation GNSS data, as well as with non-GNSS data. Section 3 gives an overview of the main identified transmitter HW impairments (i.e, ‘features’), which can separate between genuine and spoofing transmitters in RFF-based approaches. Section 4 presents the equivalent transmitter block diagrams for GNSS and spoofer signals, by emphasizing the places in the transmission payload where the various RF impairments can appear. This also shows the equivalent block diagram of the whole transmitter–channel–receiver chain and discusses the additional impairments that can be introduced by the channels and the receiver parts. Section 5 focuses on feature-extractor transforms and presents various transforms which can be employed to determine the underlying features in the received signal. Section 6 focuses on classification approaches which can be used to identify the features, after the feature-extractor transform is applied. Section 8 summarizes the main RFF solutions from the existing literature, applied on pre-correlation signals, for both GNSS and non-GNSS signals. Section 9 discusses the methods applicable to GNSS among those listed in Section 8 and offers a qualitative and comparative view of such approaches. Finally, Section 10 summarizes the open challenges in this field as well the further methodological steps to be under-taken for a designer implementing RFF algorithms based on pre-correlation GNSS data.

## 2. Problem Definition and Use-Case Example

Most of the GNSS signals use the code-division multiple access (CDMA) technique, with a received signal power around −160 dBW. This means that the received signals are usually below the noise floor. For this reason, the direct observation of the signal is in general not feasible, if not using extremely high gain antennas. Therefore, when applying RF fingerprinting it is essential to evaluate the capability of the technique to operate at low SNR.

**Figure 2:**
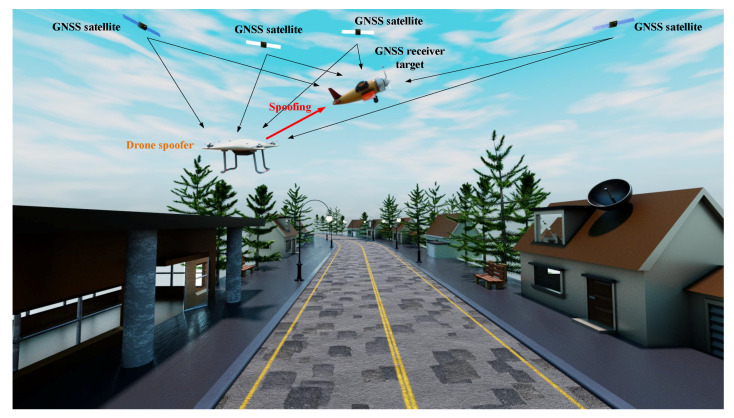
The illustration of a spoofer attacking scenario.

A spoofing scenario is illustrated in Figure 2. In this example scenario, both the drone-based spoofer and the GNSS target receiver (e.g., a civil aircraft such as a flying taxi or a rescue helicopter) receive the broadcasting GNSS signals from satellites. During the spoofing attack, the GNSS target receiver receives the spoofing signals from the spoofer as well as together with the genuine GNSS signals from sky satellites and its task is to identify and mitigate the spoofing interference for attaining optimal positioning performance. Based on the GNSS signal received from the genuine satellites on sky, the spoofer is able to create fake GNSS-like signals which it will broadcast in the air. There are many ways in which a spoofer can generate a GNSS signal, as described below, whether these involve simplistic, intermediate, and sophisticated attacks.

Figure 2 illustrates only one of the many possible scenarios one could imagine when a GNSS receiver is spoofed by one or several malicious transmitters. More details about spoofing classes and possible mitigation solutions are addressed below.

Spoofing attacks are typically split into three classes, described in detail in [11]:

- *Simplistic spoofing attacks*, such as those generated by a software defined radio (SDR) GNSS generator connected to an antenna. In this type of attack, the GNSS transmitter is not synchronized to the genuine GNSS satellites, which means that there are typically jumps in the carrier-to-noise ratios (CNR) and Doppler shifts measured at the receiver and such spoofing attacks can be identified in the pseudorange domain via various consistency checks algorithms, such as those described in [27,28,29];
- *Intermediate spoofing attacks* [30,31]: these are more complex than the simplistic attacks as they combine a GNSS generator with a GNSS receiver and are able to align the code-phase and synchronize the frequency with the signal transmitted from a genuine GNSS satellite in the sky. A replay attack or a meaconing attack with a single receiver (when the signal from a genuine GNSS satellite is captured and re-sent with a delay) is an example of such an intermediate spoofing attack;
- *Sophisticated spoofing attacks* [32]: these are the most complex spoofing attacks to mitigate, as they are an extension of the intermediate spoofing attack, where the signals received from multiple GNSS antennas (sometimes placed at different locations) are modified (e.g., through random delays and Doppler shifts) and re-transmitted in a combined manner, in such a way that the receiver is duped to believe the signals are obtained from various genuine satellites.

Spoofing attacks adversely affect the quality of positioning, navigation and timing (PNT) services of GNSS receivers, by introducing errors in the estimated PVT. For example, as shown in [31], an intermediate spoofer with a spoofer-to-signal ratio of 0 dB (i.e., equal spoofer and GNSS signal power) introducing a code delay of 0.5 chips can deteriorate the detection probability of the GNSS signal by 20% and with a code delay of only 0.25 chips, the detection probability decreases with 75% (i.e., from 100% to 25%). The spoofing impact on the good functionality of a GNSS receiver can be thus significant and it is of utmost importance to devise counter-spoofing methods, especially in life-critical applications such as aviation applications.

Current counter-spoofing methods can be classified into three main categories [11,33], according to the three GNSS-receiver domains depicted in Figure 1:

- *Pre-correlation link-level methods* relying on signal samples before the acquisition stage, i.e., on I/Q data. This is the case addressed in this paper. Such pre-correlation anti-spoofing methods are still very rare in the literature;
- *Post-correlation link-level methods* relying on the despread signal, at the output of the tracking stage for a single satellite. Examples can be found in [33,34] and they are out of the scope of this paper;
- *Navigation or system-level methods* relying on the pseudorange signals coming from all visible satellites. These are by far the most encountered anti-spoofing methods in the current literature and a few examples can be found in [27,28,29] (they are also outside the scope of this paper).

Our paper focuses on the pre-correlation spoofing identification approaches, taking as the input the I/Q raw data (at sample level) and aiming to identify, based on RF fingerprinting approaches, whether the received signal comes from a genuine GNSS transmitter or from a spoofing transmitter.

We are proposing a **four-step methodology** for the RFF-based pre-correlation spoofing detection and transmitter identification, as listed below. Each of these four steps is further detailed in Section 3, Section 4, Section 5 and Section 6.

1. **Identification of relevant features**—this step refers to first identifying the different RF ‘features’ created by the inherent hardware impairments in any transmitter. Several such features will be subsequently described in Section 3;
2. **Feature-extraction transform**—this steps refers to choosing a suitable feature-extraction transform to emphasize the selected features from the previous step. Several feature-extraction transforms are addressed in Section 5;
3. **Data pre-processing stage**—this step refers to choosing the most suitable format of saving the data at the output of the feature-extraction transform, namely as time-stamped vector data, in matrix form, as an image of certain size and number of pixels, etc. The data format selection will be influenced by the algorithms selected in the feature-classification step, as subsequently described in Section 6, as well as by the data type at the output of the feature-extraction step. For example, spectrogram-type data are also easily stored in image form, while transforms such as kurtosis or Teager–Kaiser are more suitable to be stored in a vector format;
4. **Feature classification**—this step refers to applying a selected classification methods, such as based on analytically-derived thresholds or on machine learning algorithms when training data are available, and classifying the received signal into ‘genuine’ versus ‘non-genuine/spoofer’ classes. Several feature classification approaches are discussed in Section 6. A qualitative discussion is then provided in Section 9.

**Figure 3:**
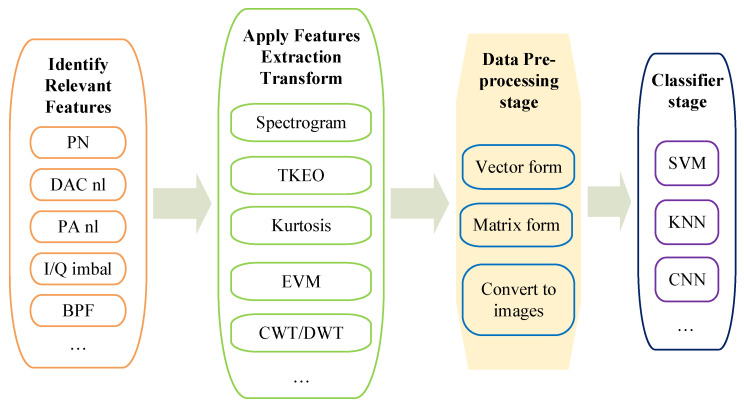
The proposed methodology for an RFF algorithm applied to GNSS pre-correlation sampled data.

The workflow of an RFF algorithm based on the aforementioned four steps is illustrated in Figure 3.

## 3. Transmitter Hardware Impairments or ‘RF Features’ Overview

A first step in building the equivalent block diagrams for a GNSS transmitter (genuine or spoofer) was to identify the possible sources of hardware (HW) impairments at the transmitter side for wideband-signal transmitters, based on works in [35,36,37,38,39,40,41,42,43] and analytical thinking. Five sources of HW impairments were identified in GNSS transmitters, as follows:

1. **Phase noise (PN)**: PN is unavoidable in any wireless transmitter, as it is introduced by the transmitter clock instabilities; atomic clocks on-board genuine GNSS transmitters are intuitively expected to have lower phase noise than the clock of spoofers and other malicious transmitters [36,37,38]. PN models are discussed in Section 3.1;
2. **Power amplifier (PA) non-linearities**: non-linearities close to the saturation region for PAs (and especially for high-power amplification needs as it is the case of GNSS transmitters) can represent an important HW feature to distinguish between different transmitters. In addition to non-linearities, possible memory effects of the PA can also create differentiating features at the transmitter. PA models are discussed in Section 3.2;
3. **I/Q imbalance**: the I/Q imbalance in a transmitter is introduced in the translation of the baseband signals to passband signals due to the facts that the phase shift is not perfectly at $90^{\circ }$ in the analogue domain and that the analogue gain is not perfectly matched for I and Q components. I/Q imbalance models are discussed in Section 3.3;
4. **Digital-to-analog converter (DAC) non-linearity**: signal distortions are also possibly produced by the non-linear DAC operation at each transmitter. DAC models are discussed in Section 3.4;
5. **Band-pass filter (BPF)** passband and out-of-band ripples: the transmitter BPF filter also puts its ‘fingerprint’ on the transmitted signal and can act as a smoother of the other HW features. BPF models are discussed in Section 3.5.

Each of these identified HW impairments is further detailed in the subsequent sub-sections.

### 3.1. PN Models

Typically, the phase noises are random noises, modelled via random time waveforms ϕ(t) and characterized by their power spectral density (PSD), denoted here via $S_{\phi }\left(f\right)$. A non-ideal local oscillator generating a waveform of amplitude A(t) at the oscillator frequency $f_{o}$ outputs a signal x(t) of the form [35]:

(1) $$x\left(t,f_{o}\right)=A\left(t\right)cos\left(2\pi f_{o}t+\phi \left(t\right)\right)$$

The PSD of the PN is typically modelled via a power law noise [44,45]:

(2) $$S_{\phi }\left(f\right)=\sum_{n_{\phi }=0}^{4}\frac{k_{n_{\phi }}}{4\pi^{2}f^{n_{\phi }}}$$

where *f* is the frequency, $k_{\phi }$ is a constant parameter of the model and $n_{\phi }=0,\ldots ,4$ are the summation parameters, defining the PN type, e.g., $n_{\phi }\in \left\{0,2\right\}$ corresponds to a white-noise model (with 0 for additive white noise sources external to the oscillator and 2 for additive white noise sources internal to the oscillator), $n_{\phi }=\in \left\{1,3\right\}$ corresponds to a flicker PN (i.e., 1 for flicker phase noise and 3 for flicker frequency noise), and $n_{\phi }=4$ corresponds to a random-walk PN.

The usually adopted model for GNSS signals is to ignore everything except the white-noise PN model at $n_{\phi }=2$ in Equation (2). In this case, the PN PSD is simplified to $S_{\phi }\left(f\right)=\frac{\sigma_{\phi }^{2}}{4\pi^{2}f^{2}}$ with $\sigma_{\phi }^{2}$ being the variance of the white noise [35]. Without a loss of generality, this white-noise PN is also the model adopted in what follows. Nevertheless, extensions to other PN PSDs are straightforward and can be easily incorporated in our model. An example of another PN PSD model can be found for example in [46] where a combination of terms at $n_{\phi }=0$ and $n_{\phi }=2$ was considered.

The on-board GNSS local oscillators are atomic clocks based on rubidium/cesium clocks [37]. Typical spoofer local oscillators have lower stability than classical atomic clocks and they rely on technologies such as oven-controlled crystal oscillator (OCXO) or temperature-controlled crystal oscillator (TCXO). This can be modelled with a lower PN variance $\sigma_{\phi }^{2}$ for genuine GNSS transmitters than for spoofers.

A typical measure of the PN PSD is through the so-called Allan variance $\sigma_{A}^{2}\left(\tau \right)$ given by [47]

(3) $$\sigma_{A}^{2}\left(\tau \right)=\frac{8}{{\left(2\pi f_{o}\tau \right)}^{2}}\int_{0}^{\infty }S_{\phi }\left(f\right)sin^{2}\left(\pi f\tau \right)df$$

Usually, it is very difficult to extract $S_{\phi }\left(f\right)$ from Equation (3), and as discussed for example in [47], there might be several $S_{\phi }\left(f\right)$ functions matching the measured $sigma_{A}^{2}\left(\tau \right)$. Nevertheless, for the purpose of RFF, we are not interested in measuring the exact $S_{\phi }\left(f\right)$, but we only consider it as one of the HW features at the transmitter, with the assumption that the spoofer and the genuine GNSS transmitters have different PSDs $S_{\phi }\left(f\right)$.

### 3.2. PA Non-Linearity Models

The power amplifier is an important element in the wireless communications system, and its non-linearity behaviour varies from device to device. It is expected that PA non-linearities can also be used as differentiating features between GNSS satellite transmitters and spoofers or jammers, due to the fact the GNSS PAs are high-cost high power amplifiers (HPA), such as solid state power amplifiers (SSPA) or a travel-wave tube amplifier (TWTA) [39], while non-genuine GNSS transmitters typically have low-power amplifiers (LPA) [40]. The highest PA power efficiency is achieved at the saturation point, where heavy non-linearity occurs in all PA models [48].

**Figure 4:**
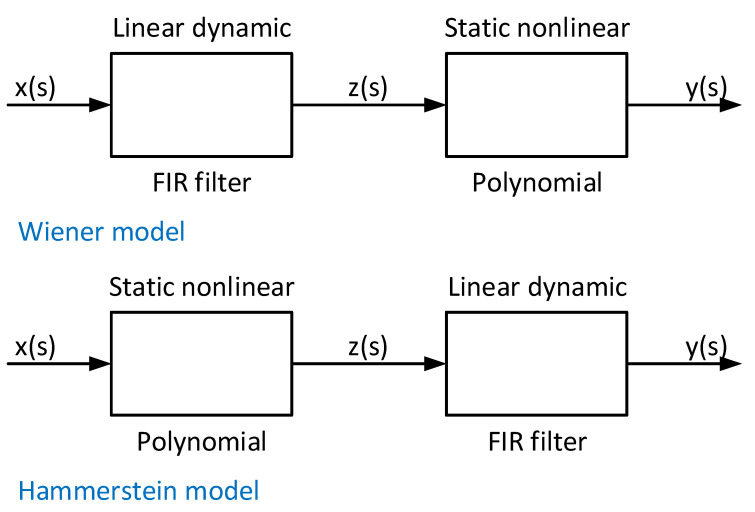
The block diagram for the Wiener model and Hammerstein model.

There are typically two classes of models for PA non-linearities [49]: the memoryless non-linear models and the non-linear models with linear memory. The memoryless non-linear model of a system with input x(t) and output y(t) (assuming a continuous-time model) is given by the $L_{th}$ order polynomial:

(4) $$y\left(t\right)=\sum_{l=1}^{L}\alpha_{l}x^{l}\left(t\right)$$

where $\alpha_{l},l=1,\ldots ,L$ is the $l_{th}$ coefficient of a PA non-linearity of order *L*. When the wideband signals pass through the power amplifier, the bandwidth of signals is not negligible compared with the inherent bandwidth of the amplifier, and therefore a frequency-dependent behaviour occurs. This behaviour is called a memory effect. Regarding the non-linear model with linear memory, the two most encountered models are the Wiener model and the Hammerstein model, as described in [49]. We illustrated these two models in Figure 4. The corresponding mathematical expressions (this time in the discrete-time domain) are, respectively:

Wiener model:

(5a) $$\begin{array}{ccc} \; & \; & \;y_{Wiener}\left(s\right)=\sum_{n=0}^{N}c_{n}{\left[\sum_{q=0}^{Q-1}h\left(q\right)x\left(s-q\right)\right]}^{n} \end{array}$$

Hammerstein model:

(5b) $$\begin{array}{ccc} \; & \; & \;y_{Hammerstein}\left(s\right)=\sum_{q=0}^{Q-1}h\left(q\right)\left[\sum_{n=0}^{N}c_{n}x^{n}\left(s-q\right)\right] \end{array}$$

where *s* is the sample index (assuming the x(t) signal was sampled at a sampling rate $1/T_{s}$, namely at $t=s/T_{s}$ time instants), h(q) denotes the *q*-th coefficient of a finite impulse response (FIR) filter, and $c_{n}$ denotes the $n_{th}$ order coefficient in the polynomial memory model.

Due to the difficulties of estimating the coefficients for FIR filters in both the Wiener and Hammerstein model, the memory polynomial [50] has become a popular model for the behaviour of power amplifiers. The expression of MP is given by [50],

(6) $$y_{MP}\left(n\right)=\sum_{k=0}^{K-1}\sum_{m=0}^{M}a_{km}x\left(n-m\right){\left|x(n-m)\right|}^{k}$$

where $a_{km}$ are the model parameters. In our work, we used the memory polynomial to model PA in navigation payload with user-defined model parameters $a_{km}$ which were considered different for each transmitter (i.e., satellite and spoofer transmitters).

In order to maximize the power efficiency and the lifespan of the satellite payload, the GNSS signals are usually designed to exhibit a (quasi) constant complex envelope. For instance, this is achieved by including an inter-modulation product among the signal components. For this reason, it is reasonable to expect that the PA non-linearities will not significantly distort the genuine GNSS signals. This might not hold for many spoofing signals, which may simplify the signal generation by only emulating some of the signal components and/or omit the inter-modulation product. However, it shall be noted that a spoofer usually needs to generate low-power levels, hence it is easier to ensure linearity with LPA. The fact that the spoofer needs to transmit at a lower power than the GNSS transmitters is due to the fact that spoofers are usually within the range of a few tens of meters to a few km away from the GNSS receivers, while GNSS satellites are at more than 20,000 km away from the receivers.

### 3.3. I/Q Imbalance Models

During the baseband-to-passband conversion, the I and Q components ($x_{I}\left(t\right)$ and $x_{Q}\left(t\right)$) at the transmitter can be modelled via [41,42]:

(7) $$\begin{array}{c} x_{I}\left(t\right)=A\left(t\right)cos\left(2*pi*f_{c}\right)\\ x_{Q}\left(t\right)=g_{IQ}A\left(t\right)sin\left(2*pi*f_{c}+\phi_{IQ}\right) \end{array}$$

where A(t) is the baseband amplitude, $f_{c}$ is the passband carrier frequency, $g_{IQ}$ is the I/Q amplitude imbalance factor, also known as the gain imbalance factor [42] and measured typically in dB, and $\phi_{IQ}$ is the I/Q phase imbalance factor, also known as quadrature skew factor [42]. Above, the PN effect was ignored for clarity purposes. The imbalance factors $g_{IQ}$ and $\phi_{IQ}$ are transmitter-dependent constants and it is expected that a genuine GNSS transmitter would have lower absolute values $|g_{IQ}|$ and $|\phi_{IQ}|$ than a spoofer. For a perfect transmitter, without any I/Q imbalance, one would have $g_{IQ}\left[dB\right]=0$ and $\phi_{IQ}=0$. Imperfect transmitters have been studied for example in [42], based on multipurpose universal software radio peripheral (USRP) as those that may be used by a Software Defined radio (SDR) spoofer and values below 1 dB and below 8 degrees have been estimated for $|g_{IQ}|$ and $|\phi_{IQ}|$ values, respectively.

### 3.4. DAC Models

Based on [43], the DAC model is given by

(8) $$y\left(t\right)=x\left(t\right)+x^{HQ}\left(t\right)+x^{CM}\left(t\right)+x^{VQ}\left(t\right)$$

where y(t) is the output continuous-time signal, x(t) is the input continuous-time signal and the corresponding discrete-time form is x[n], $x^{HQ}\left(t\right)$ is the horizontal quantization additive effect, $x^{CM}\left(t\right)$ is the clock additive effect, and $x^{VQ}\left(t\right)$ is the vertical quantization additive effect. The horizontal quantization additive effect $x^{HQ}\left(t\right)$ is given by

(9) $$x^{HQ}\left(t\right)=\sum_{n=-\infty }^{\infty }x\left[n\right]g\left(\frac{t-nT_{g}}{T_{g}}\right)-x\left(t\right)$$

where $T_{g}$ is a constant generation period, g(t) is a unitary pulse function:

(10) $$g\left(t\right)=\left\{\begin{array}{cc} 1, & 0\leq t\leq 1\\ 0, & \mathrm{elsewhere} \end{array}\right.$$

The clock additive effect $x^{CM}\left(t\right)$ is:

(11) $$x^{CM}\left(t\right)=\sum_{n=-\infty }^{\infty }x\left[n\right]h_{n}\left(t-nT_{g}\right)$$

where $h_{n}\left(t\right)$ yields to:

(12) $$h_{n}\left(t\right)=-\mathrm{sign}\left(\Delta_{n}\right)g\left(\frac{t-nT_{g}}{\Delta_{n}}\right)+\mathrm{sign}\left(\Delta_{n+1}\right)g\left(\frac{t-\left(n+1\right)T_{g}}{\Delta_{n+1}}\right)$$

where $\Delta_{n}$ is a time amount. For example, based on (10), $g\left(\frac{t}{\Delta_{n}}\right)$ has a rising edge at time instant zero and a falling edge at time instant $\Delta_{n}$. By assuming the nearest voltage level that DAC could provide for x[n] is $\hat{x}\left[n\right]$, the vertical quantisation additive effect is:

(13) $$x^{VQ}\left(t\right)=\sum_{n=-\infty }^{\infty }\left\{\hat{x}\left[n\right]-x\left[n\right]\right\}\cdot \left[g\left(\frac{t-nT_{g}}{T_{g}}\right)+h_{n}\left(t-nT_{g}\right)\right]$$

**Figure 5:**
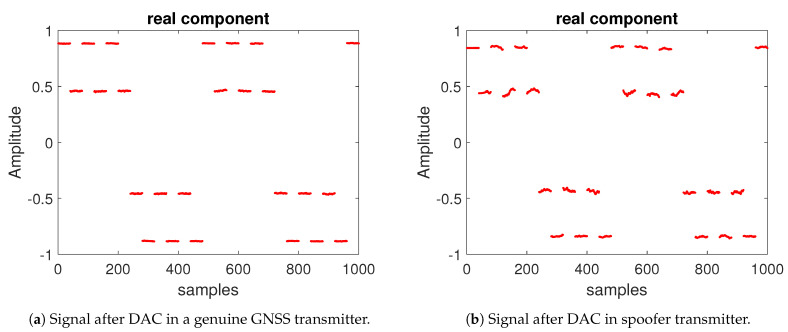
Examples of DAC characteristics at the transmitter, for a genuine (**a**) and a spoofer (**b**) transmitter.

Here, we demonstrate two examples in Figure 5a,b to illustrate the effect of DAC in different transmitters. These two examples are given for the in-phase components of the signal. Clearly, the distortions existing in spoofer DAC are heavier than that in a genuine GNSS transmitter.

### 3.5. BPF Models

The band-pass filter (BPF) is equipped at transmitters to filter out undesired non-central frequencies signals. In this work, we model BPF using a finite impulse response (FIR) filter. A general form of an FIR filter output y[n] can be given by

(14) $$y\left[n\right]=\beta_{0}x\left[n\right]+\beta_{1}x\left[n-1\right]+\cdots +\beta_{k}x\left[n-k\right]+\cdots +\beta_{K}x\left[n-K\right]$$

where $\beta_{k}$ is the $k_{th}$ impulse response, *K* is the order of the filter.

**Figure 6:**
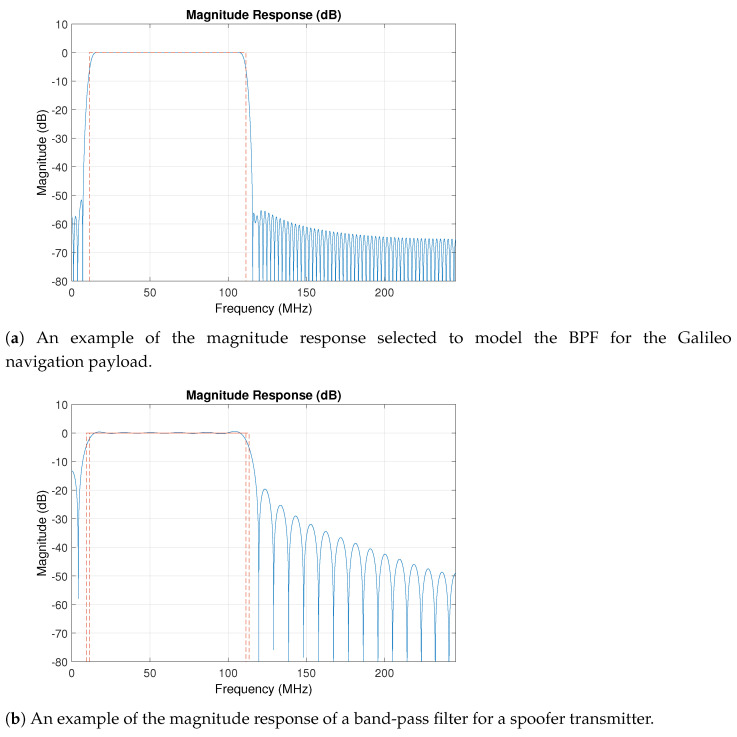
Examples of characteristics of the band-pass filter at the transmitter for a genuine (**a**) and spoofer (**b**) transmitter.

We use the window design method for the genuine GNSS transmitter BPF and the least squares method for spoofer transmitter BPF. An example of BPFs used for a genuine GNSS transmitter versus a spoofer transmitter is shown in Figure 6a,b, respectively. The exact parameters of the filters used in the genuine GNSS transmitters are not known, however, without loss of generality, the assumption here is that the passband and stop-band ripples of a BPF for a genuine transmitter are smaller than those for the BPF of a spoofer. This is expected to be more evident for spoofers based on SDR, which generally include configurable BPFs.

## 4. Equivalent Block Diagrams for GNSS and Spoofing Signals

Section 3 identified the main sources of the transmitter feature. This section will present, to the best of our knowledge for the first time in the literature, two equivalent simplified models of a genuine GNSS transmitter and a spoofer GNSS transmitter, by taking into account all five HW impairments identified and discussed in the previous section. These equivalent models will serve as the bases for addressing RFF in the context of GNSS, as they clearly identify the places of various HW features and point out situations where the same type of feature (e.g., phase noise) can affect multiple blocks. In order to build these equivalent transmitter block diagrams, we gathered information from the Galileo standards and manufacturer brochures, e.g., as in [51] and from software-defined radio GNSS transmitter sheets such as those in [52].

### 4.1. Equivalent Transmitter Block Diagrams

**Figure 7:**
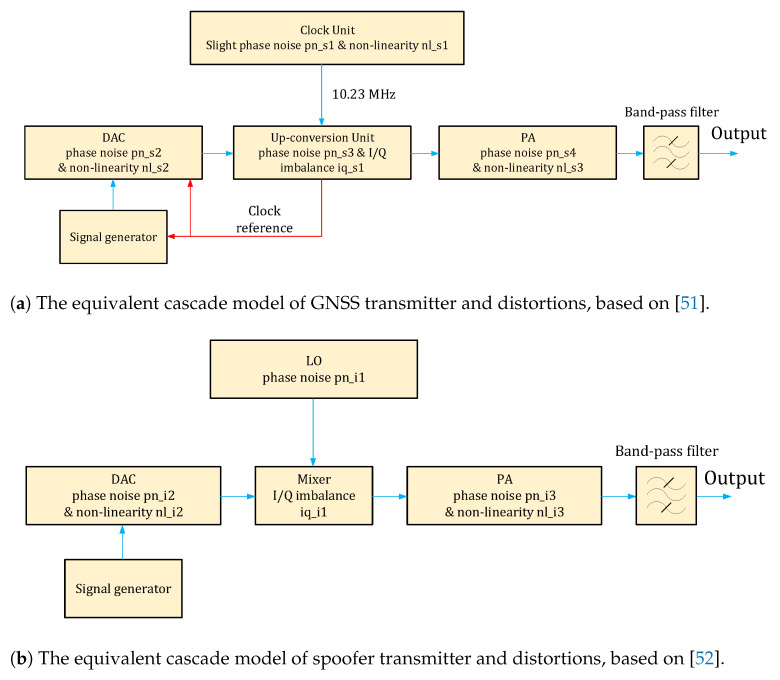
The diagrams of an equivalent model for GNSS and spoofer transmitter. In GNSS transmitters, all distortions are indexed with s*; in a spoofer transmitter, all distortions are indexed with i*.

The equivalent block diagrams of a GNSS (e,g., Galileo) satellite transmitter and of a spoofer GNSS transmitter are depicted in Figure 7a,b, respectively. These summarizing block diagrams help in identifying at a glance the places where the different HW impairments discussed in Section 3 appear. For example, phase noises can appear in each of the transmitter blocks, such as the clock unit, Digital-to-Analog Converter (DAC), up-conversion unit/mixer units and power amplifier. I/Q imbalances are typically only present in the up-conversion unit/mixer units. Non-linearities can appear in the DAC and PA units. Different blocks have different noise levels: for example, the phase noise pn_s1 (s stands for satellite here) from the clock unit is not the same phase noise as in the up-conversion unit (phase noise pn_s2), etc. Moreover, the phase noise pn_s1 from the GNSS transmitter is different from the phase noise pn_i1 from spoofer (i stands for interferer, here), and the same is valid for all the different transmitter ((s1, s2, s3, …) and spoofer (i1, i2, …) features depicted in Figure 7a,b. The non-linearity nl_s1 effect in the Galileo clock unit (Figure 7a) appeared due to the additional DAC units employed in the Galileo clock unit [51]. Such additional DACs are, however, unlikely to be used in a spoofer, and thus the local oscillator (LO) of a spoofer ( Figure 7b) does not exhibit additional non-linearity effects.

The GNSS power amplifier is typically an HPA, while the spoofer power amplifier is typically an LPA, as discussed in Section 3. The levels of various transmitter impairments are not known for GNSS transmitters and need to be learnt via the RFF feature extractors and classifiers discussed next, and based on training data. High-quality training data would need GNSS samples at various sampling rates (i.e., corresponding to both low-end and high-end receivers), for the duration of several milliseconds for each training sequence, and typically thousands of training sequences for robust RFF results. This may represent one of the main challenges or bottlenecks of RFF approaches at the pre-correlation level: for example, 2 ms of data sampled at a moderate sampling rate of 24 Mbps has 24,000 complex-valued samples per each sequence in the training data. Assuming 1000 sequences in the training database and data saved on 8-bit per real sample, this would require 0.48 GB of data in each training sequence. The 2 ms of data pieces per training sequence was shown as an example. We expect that several milliseconds of observations of I/Q raw data will be needed. As a rule of thumb, GNSS signal acquisition is usually performed using at least 10 ms. The needed size for the training databases increases with the increased processing time, with the increased sampling rate, and with the increased amount of sequences in the training database. Through some of the feature-extraction methods discussed in Section 5, one can reduce the dimensionality of the data, for example using images instead of matrices, or applying principal component analysis (PCA) methods to reduce the data dimensionality. More about PCA will be discussed in Section 6.

### 4.2. Equivalent Block Diagram of the Full Transmitter-Channel-Receiver Chain

**Figure 8:**
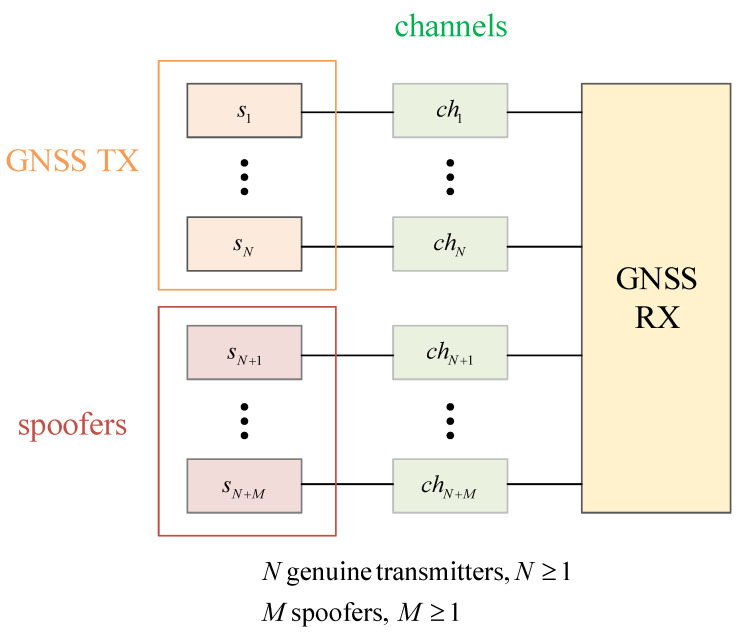
The illustration of the EVM principle. The blue arrow denotes the transmitted symbol, the yellow arrow denotes the received symbol, the blue arrow denotes the estimate, and the crimson arrow denotes the estimation error.

Figure 8 shows the equivalent full transmission chain of a generic system with *N* genuine GNSS transmitters and *M* spoofers, N≥1,M≥1. Assuming that spoofers (if more than one) are placed at different locations, the wireless channel experimented by each of the genuine and non-genuine transmitters will exhibit different multipath and fading profiles, as well as different noise levels. In this generic example, there will be N+M different wireless channels, which can typically be assumed to be non-correlated. A typical channel impulse response $h_{i}\left(t\right),i=1,\ldots N+M$ can be modelled via a tapped-delay line with $L_{i}$ multipaths via

(15) $$h_{i}\left(t\right)=\sum_{l=1}^{L_{i}}\alpha_{i,l}\delta \left(t-l\tau_{i,l}\right)$$

where $\alpha_{i,l}$ are the complex channel coefficients of the *l*-th path of the *i*-th channel, and $\tau_{i,l}$ are the multipath delays of the *l*-th path of the *i*-th channel. Above, δ(t) is the Dirac pulse. Clearly, such a channel acts as a finite impulse response (FIR) filter which is likely to smooth out some of the transmitter HW features.

A signal $s_{i}\left(t\right),i=1,\ldots N+M$ originated from a genuine GNSS transmitter (i=1,…N) or from a spoofer (i=N+1,…N+M) will reach the receiver antenna in the combined form r(t):

(16) $$r\left(t\right)=\sum_{i=1}^{N+M}\sum_{l=1}^{L_{i}}\alpha_{i,l}s_{i}\left(t-l\tau_{i,l}\right)+\eta_{i}\left(t\right)$$

where $\eta_{i}\left(t\right)$ is the additive noise corresponding to the *i*-th channel. Typically, $\eta_{i}\left(t\right)$ is modelled as the Gaussian noise of a zero mean and $\sigma_{i}^{2}$ variance, and the overall channel variance $\sum_{i=1}^{N+M}\sigma_{i}^{2}$, as well as the transmitted signal power, which will determine the carrier-to-noise ratio (CNR) at the receiver. The impact of the channel effects on the RFF have been reported as either insignificant or as negative in the literature so far, meaning that the transmitter features were either found to be invariant to the type of channel (static versus fading, multipath versus single path, etc.) [53,54] or to adversely affect the transmitter features, by smoothing them out [55]. However, very few studies, to the best of our knowledge, addressed the impact of channel impairments on the RFF, and to date, all have been performed in a non-GNSS context. For example, the studies on [53] were done for WiFi signals, the studies in [54] were for Zigbee signals, and the studies in [55] were for 3G cellular signals. Therefore, more simulation-based and measurement-based experiments are needed in order to fully understand the channel effects on RFF in GNSS and this remains one interesting research challenge.

Furthermore, the receiver from Figure 8 also has its own HW elements such as front-end filtering, analog-to-digital (ADC) conversion, local oscillators, and power amplification, and each of these elements will act as additional distortions to the individual transmitter features, as they will be common to all signals $s_{i}\left(t\right)$ found in the received signal r(t) (see Equation (16)). As shown in [3], the same GNSS data from GNSS satellites collected with two different antennas give different fingerprints. This means that, in order to be able to fully identify a GNSS transmitter, one should be able to remove the receiver front-end features from the analysis. For example, one could try to model the behaviour of a certain type of receiver (e.g., USRP, commercial GNSS receiver) and a certain antenna type (e.g, Talysman, Zenith, etc.) and try to compensate the fingerprint it produces via some equalization-like functions. No such models exist in the current literature, according to the best of our searches, and this also remains a topic of open investigation. Moreover, the impact of the receiver sampling rate on RFF accuracy remains to be addressed in the GNSS context. Some studies of the effect of quantization and sampling rates on RFF in the context of non-GNSS signals can be found in [56] (for WiFi signals) and [57] (for BLE signals) and the current understanding is that, typically, higher sampling rates give better RFF accuracy. Such findings are still to be confirmed in the GNSS context.

## 5. RF Feature Extractors

Section 3 gave an overview of the main RF features that a wireless transmitter can have. The question addressed in this section is how to identify such features, or, more precisely, what feature-extraction transforms T(·) are available from the literature.

### 5.1. Error Vector Magnitude (EVM)

The error vector magnitude is a time-domain transform that measures how far the estimated symbols at the receiver side may deviate from the true symbols. I/Q imbalance, thermal noise, in- and out-of-band leakage, and phase noise are all causes that can degrade the EVM metric, thus EVM has the potential to be a good feature-extractor transform to capture hardware impairments from the received signals.

**Figure 9:**
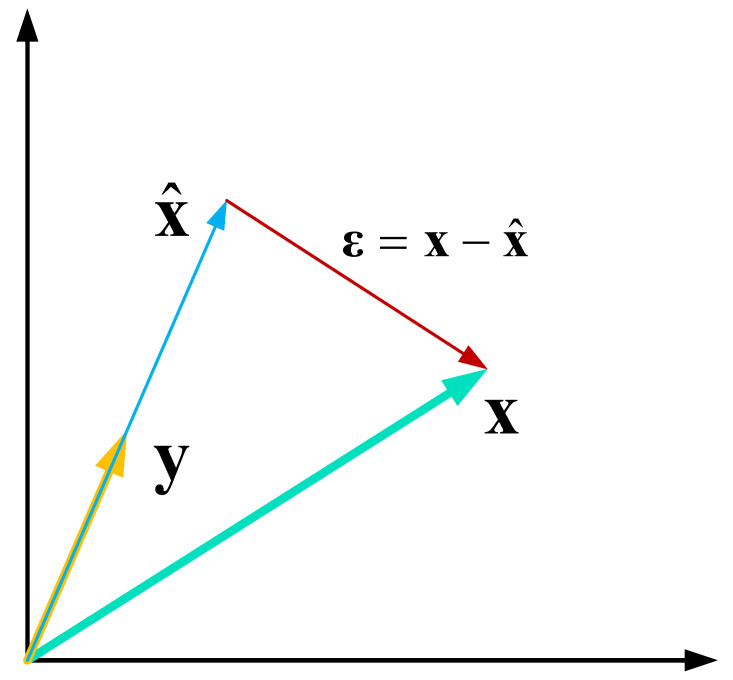
The illustration of EVM principle. The blue arrow denotes the transmitted symbol, the yellow arrow denotes the received symbol, the blue arrow denotes the estimate, and the crimson arrow denotes the estimation error.

In general, EVM is applied in the context of demodulated signals, as follows: let us assume that a symbol x is transmitted, and that at the receiver, a symbol y is received. The receiver estimates (e.g., via decoding process) the symbol $\hat{x}$. Therefore, the estimation error ϵ is: $\epsilon =x-\hat{x}$, as depicted in Figure 9. The EVM of the symbol x is defined as

(17) $${\mathrm{EVM}}_{x}≜\frac{{∥\epsilon ∥}_{2}}{{∥x∥}_{2}}$$

where ${∥\cdot ∥}_{2}$ is the Euclidian norm.

When the input to the EVM transform is the I/Q sampled data, one can apply the EVM as follows: x is a complex-valued sequence of an ideal GNSS signal (i.e., without any distortions); it can be generated, for example, via a GNSS signal generator; $\hat{x}$ is the received signal (genuine or spoofer) at the I/Q level. Then, the EVM based on pre-correlation data measures the discrepancy between an ideal GNSS signal and the received signal. Under the hypothesis that the spoofer transmitter non-idealities will be further away from the ideal case x than the GNSS transmitter non-idealities, then the EVM of a genuine GNSS signal is expected to be smaller than the EVM of a spoofer.

**Figure 10:**
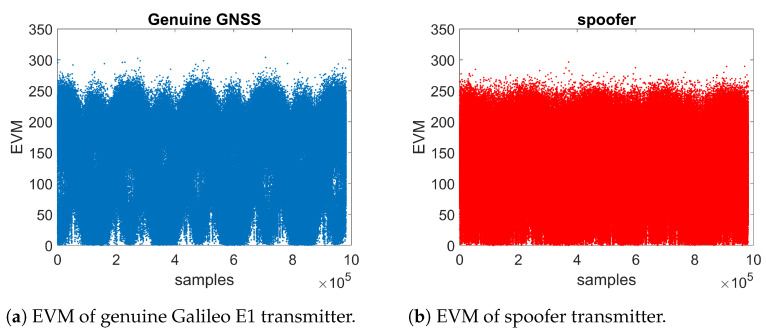
Illustrative example of EVM applied on pre-correlation data, in the absence of channel and receiver effects.

Figure 10a,b show two illustrative examples of EVM outputs for genuine GNSS transmitter and spoofer, respectively (both using Galileo E1 signal specifications and based on a software simulator built by us). The EVM results for the genuine Galileo E1 transmitter and spoofer have visible differences, with EVM values for the spoofer being, on average, slightly higher than those for the Galileo signal, as predicted by the theory. The examples in Figure 10a,b are based on a very high CNR of 100 dB–Hz, for illustrative purposes. At lower CNRs, such differences are no longer visible to the naked eye, but they still have some potential to be captured by a machine learning algorithm, for example.

### 5.2. Kurtosis

Kurtosis is a measure of the Gaussian behaviour of a random variable and it is defined as

(18) $$T_{kurtosis}\left(r\left(n\right)\right)=E\left({\left(\frac{r(n)-E(r(n))}{std(r(n))}\right)}^{4}\right)$$

where r(n) is the complex sampled signal (sampled at sampling times $nT_{s}$, with $T_{s}=1/f_{s}$ being the sampling interval, and $f_{s}$ the sampling frequency); E(·) is the expectation operator, and std(·) is the standard deviation operator. For Gaussian-distributed sequences r(n), $T_{kurtosis}\left(r\left(n\right)\right)$ is close to level 3. For non-Gaussian distributed sequences, this value is higher or larger than 3. Kurtosis was one of the feature extractors selected in our simulations.

**Figure 11:**
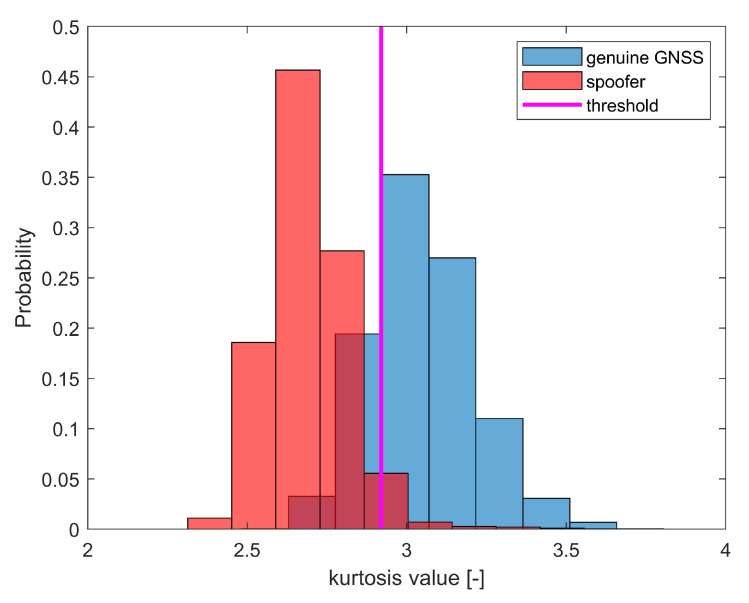
Example of the Galileo E1 and spoofer histograms when kurtosis is applied as a feature extractor.

An example of a histogram for the kurtosis results of genuine GNSS transmitter and spoofer is shown in Figure 11. The magenta line represents the threshold to differentiate the spoofer from a genuine GNSS transmitter. It is typically expected that the received GNSS signals in the pre-correlation domain are Gaussian (see blue histogram from Figure 11), due to the fact that the pre-correlation data are dominated by the thermal noise. In the presence of a strong spoofer, this Gaussian property may be lost, due to the fact that spoofer power might become the dominant one.

### 5.3. Teager–Kaiser Energy Operator (TKEO)

The Teager–Kaiser energy operator (TKEO) is a transform which can estimate the instantaneous energy of a signal, and thus may uncover features that are distinguishable in power or energy. The TKEO transform $T_{TKEO}$ of a complex signal r(n) is defined as [58]

(19) $$T_{TKEO}\left(n\right)={\left|r\left(n\right)\right|}^{2}-\frac{1}{2}\left(r^{*}\left(n+1\right)r\left(n-1\right)+r\left(n+1\right)r^{*}\left(n-1\right)\right)$$

where r(n) is the complex sampled signal and $r^{*}\left(n\right)$ is the conjugate of r(n).

TKEO has been previously used in the context of RFF in GNSS in [3] with promising results. It is also one of the feature extractors selected in our study.

### 5.4. I/Q Data Spectrograms and Other Short-Time-Short-Frequency (STSF) Transforms

The short-time Fourier transform (STFT) $T_{STFT}$ is simply a Fourier transform within a window (i.e., short time); and the discrete STFT over a window of $N_{w}$ samples of the received signal r(·) is given by

(20) $$T_{STFT}\left(f,m\right)=\sum_{n=1}^{N_{w}}r\left(n\right)w\left(n-m\right)e^{-j2\pi fn}$$

where *m* is the time sample index, the r(n) is the complex sampled signal, containing the I and Q components (r(n)=I(n)+jQ(n)), *f* is the frequency, and w(·) is a time window (e.g., Hamming, Hannig, etc.). The spectrogram $T_{Spectrogram}$ is squared absolute value of the STFT transform, namely:

(21) $$T_{Spectrogram}\left(f,m\right)={\left|T_{STFT}\left(f,m\right)\right|}^{2}$$

Clearly, $T_{Spectrogram}\left(f,m\right)$ and $T_{STFT}$ are two-dimensional frequency-time transforms and can be stored both as a matrix and in image form. We investigated both approaches and found that by storing the spectrogram into an image form, we obtained more accurate results than by operating with the matricial form.

**Figure 12:**
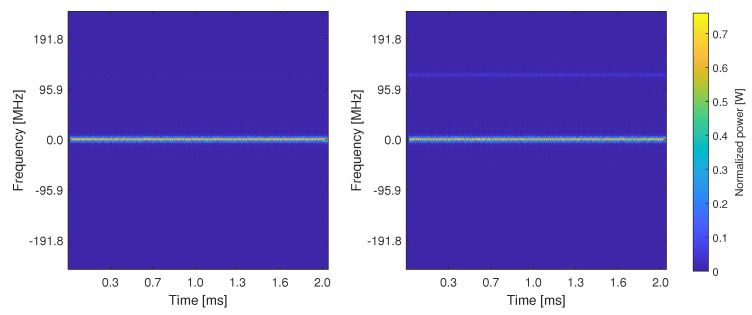
An example of spectrogram-based feature extraction. The left-hand figure is a spectrogram of genuine GNSS (Galileo E1) transmitter, the right-hand figure is a spectrogram of spoofer (Galileo E1) transmitter.

Figure 12 shows the comparisons of spectrogram-based results between a genuine Galileo E1 transmitter and a spoofer also based on Galileo E1 signal characteristics. The results are based on our in-house Matlab-based simulator, based on the block diagrams in Figure 7a,b and at a very high carrier-to-noise (CNR) ratio of 100 dB–Hz, in order to be able to also identify (for illustration purposes) the different HW features by the naked eye. The results are shown in the absence of channel and receiver effects. It can be seen in Figure 7a,b that there exist visible differences between these two images, e.g., the spectrogram of spoofer I/Q data has one extra line on the upper half of the image compared to the spectrogram of the genuine Galileo I/Q data. The underlying models of the HW features used in our simulator for the genuine and spoofer transmitters were based on the assumptions that phase noises and I/Q imbalances were weaker for a genuine signal than from the spoofer signal. The PA non-linearity models were based on [59], by picking two different PA non-linearity models from there to characterize the spoofer and the genuine GNSS transmitter.

### 5.5. Wavelet Transforms

A wavelet transform decomposes an incoming signal into some ‘coarse’ and ‘fine’ coefficients, based on shifted and scaled versions of a so-called ‘mother wavelet’ function. Unlike the Fourier transform that cannot offer compact support in both the time and frequency domains, a wavelet transform can offer a compact/bounded support in both tome- and wavelet-domains. Wavelet transforms have been extensively used in watermarking and image-processing applications, and have been reported to be able to identify ‘hidden’ features; thus, they look like relevant feature extractors for RF fingerprints. Wavelet transforms, in the context of RF fingerprinting, have been previously used, for example in [25,60,61]. The work in [25,60] was only focusing on narrowband signals, in contrast to GNSS. The work in [61] used GNSS simulation-based signals, but only focused on a few simplified transmitter HW impairments. While the work in [61] showed some limited promising results with the discrete wavelet transforms in the context of RFF, our further investigations with more realistic transmitter models as described in Section 3 and Section 4 did not show any improvement by using a wavelet transform instead of a spectrogram. Wavelet transforms have an increased complexity compared to other feature-extraction transforms because they output two pairs of complex coefficients (the coarse and fine-approximation coefficients); by distinction, for example, the spectrogram only has one complex output sequence.

## 6. RF Feature Classifiers

Feature classification methods can be typically split into two main classes: (i) methods based on thresholding or the direct sorting of the outputs of the feature extraction stage; and (ii) methods based on machine learning (ML) classifiers. The second category was by far the category most encountered in RF fingerprinting, as shown previously in Table 1.

### 6.1. Threshold-Based Classification

The threshold-based classification is also known as a traditional hypothesis testing and can be implemented through well-known algorithms such as likelihood ratio testing (LRT) or Gaussian likelihood ratio testing (GLRT) [83,84]. The traditional hypothesis testing problem is a problem of distinguishing between two hypotheses, namely $H_{0}$ and $H_{1}$:

(22) $$\begin{array}{c} \left\{\begin{array}{ccc} H_{0} & & \mathrm{spoofer}\mathrm{is}\mathrm{absent}\\ H_{1} & & \mathrm{spoofer}\mathrm{is}\mathrm{present} \end{array}\right. \end{array}$$

If the feature-extraction transform outputs scalar or vector values (instead of N-dimensional matrices with N≥2), a classification can be envisaged via a simple threshold, for example, by comparing the scalar value or the vector statistics (mean, minimum, maximum, median, etc.) to a certain pre-defined threshold. If the data are in N-dimensional form, then LRT/GLRT methods with Gaussian multivariate modelling can be employed.

The challenging part in this approach is choosing a suitable threshold, when no a priori knowledge about the genuine and spoofing signals is available. Such a threshold can be determined based on theoretical assumptions (e.g., kurtosis transform is known to be close to 3 for Gaussian-distributed variables) or by using an initial training base with genuine and spoofing signals and derive a threshold based on the training database. Another challenge in this threshold-based approach is that most of the transmitter features are ‘hidden’ and not distinguishable through classical hypothesis testing, as the probability distribution functions of $H_{0}$ and $H_{1}$ hypotheses from Equation (22) would overlap.

In our work, we used the optimal false alarm rate from ML-based classification to calculate the detection rate. By comparing this detection rate with the optimal one from ML-based classification, we evaluate the performance of the threshold-based method.

### 6.2. ML-Based Classification

Machine learning (ML) methods have been widely used in the literature as methods of classification in RF fingerprinting approaches or for transmitter identification and authentication (see the references from Table 1). Typically, three main classes of ML approaches are encountered, namely: unsupervised learning (k-means, fuzzy k-means, etc), supervised learning (e.g., kNN, SVM, random forest, gradient boosting, etc.), and reinforcement learning (e.g., Markov decision processes, etc.). In addition, deep learning methods, such as CNN, can be applied typically both in a supervised or unsupervised manner. The fact that the data are not annotated or labelled in unsupervised approaches makes the unsupervised approaches less useful than the supervised ones in the context of RFF, where one would like to have the exact labels of the genuine transmitters. Moreover, reinforcement learning methods are typically rather complex and rely on harnessing additional data from the environment. They have not been studied yet in the context of RFF for GNSS to the best of the authors’ knowledge and are highly unlikely to work with GNSS pre-correlation data as their complexity combined with the huge amount of pre-correlation data to be processed will be prohibitive. A recent, not yet peer-reviewed work on reinforcement learning (i.e., a policy gradient method) with RFF for an ADALM-PLUTO software defined radio (SDR) can be found in [85], but the focus in there was to apply reinforcement learning to enhance the spoofer capabilities in the context of a quadrature phase shift keying (QPSK) communication systems, not to identify the spoofer. For these reasons, only the supervised and deep learning approaches have been investigated to date in the context of RFF and these are also the ones we will briefly describe in the next sub-sections.

#### 6.2.1. kNN Classifier

**Figure 13:**
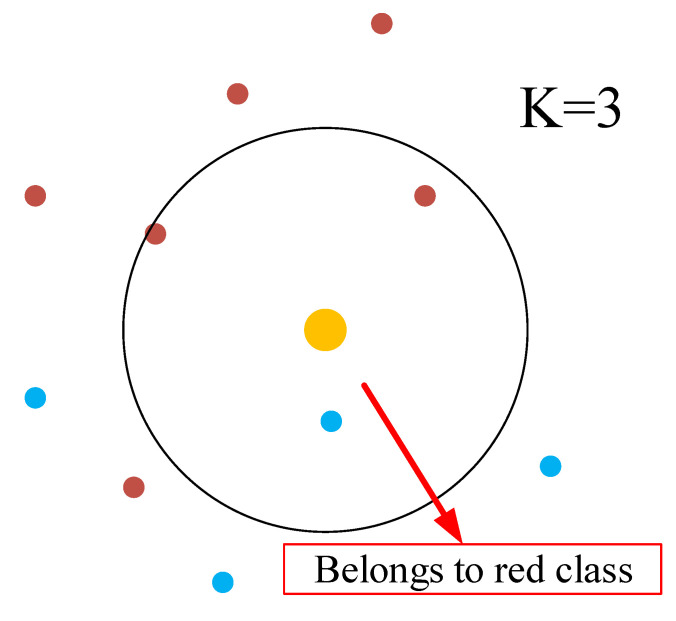
An example of KNN for three nearest neighbours.

The kNN classifier is the most used classifier from the class of unsupervised ML approaches. The principals behind it are simple: for every sample, it will look at the k nearest neighbours, and the class of this sample will be determined by the class of the majority in the nearest neighbours. Figure 13 presents an example when the nearest neighbours are three: the three nearest neighbours of a testing yellow dot are two red dots and one blue dot, and as a result, the yellow dot is determined as a red class.

**Figure 14:**
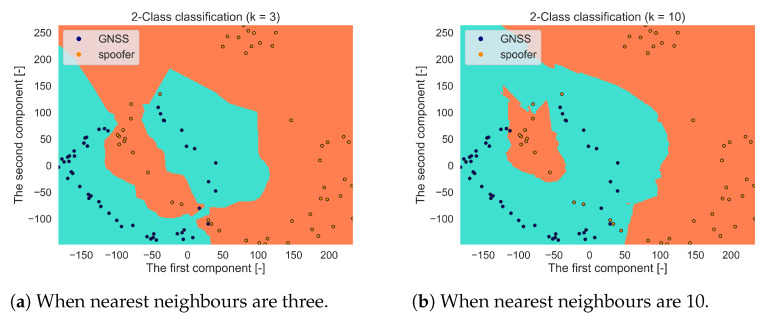
An example based on Galileo E1 and spoofer simulated data: the first two principal components of PCA of spectrogram images are classified by KNN under a different number of nearest neighbours: 3 (**left**) and 10 (**right**).

Figure 14a,b demonstrate the impact of a different number of nearest neighbours on the boundary of two classes (a spoofer and a Galileo E1 signal). A large number of nearest neighbours may lead to an over-fitting problem while an insufficient number of nearest neighbours degrades the classification performance.

#### 6.2.2. SVM Classifier

As the problem we address here is a classification problem with two classes: spoofer absent (or $H_{0}$ hypothesis) versus spoofer present (or $H_{1}$ hypothesis), the most encountered ML classifier for a two-class problem is the support vector machine (SVM), as SVM is designed to maximize the margin between classes in such a two-class case. The SVM classifier could be versatile by using a kernel trick. Considering 2D points (x,y), here, we list several popular kernels k(x,y):

- Linear kernel: k(x,y)=x·y;
- Polynomial kernel: $k\left(x,y\right)={\left(x\cdot y\right)}^{d}$, *d* is the exponent;
- Sigmoid kernel: k(x,y)=tanhax·y+b, a>0 and b<0;
- Gaussian kernel (also known as an rbf kernel): $k\left(x,y\right)=e^{\left(-\frac{{∥x-y∥}^{2}}{2\sigma^{2}}\right)}$.

Typically, a Gaussian kernel takes best into account the irregular boundary in the I/Q GNSS datasets. As the dimensions of raw I/Q data are typically huge, some forms of dimensionality reduction can be typically employed. One such form is known under the name of **Principal components analysis (PCA)**.

PCA is a common method to pre-process data for the purpose of reducing the dimension of the target dataset before the classifications. The first few principal components implies the most dominant features existing in the dataset, whilst using PCA is an effective way to improve the classification performance.

**Figure 15:**
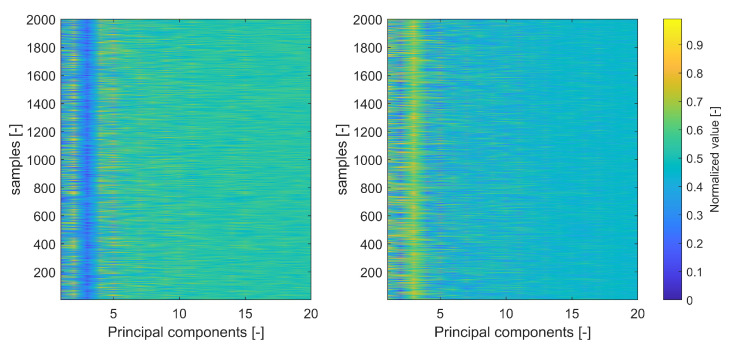
Comparisons between the PCA results in spectrogram images of GNSS (**left**) and spoofer (**right**). The values in the colour bar represent the amplitude levels of the PCA coefficients.

For example, Figure 15 demonstrates the first 20th components in the spectrogram images of a Galileo E1 and a spoofer (also based on Galileo E1 signal specifications), respectively. The plots are shown for a very high CNR level (100 dB–Hz) for illustration purposes. The PCA levels are clearly distinct in the two plots of Figure 15, pointing out the fact that the various transmitter HW features can indeed differentiate between the transmitter types to some extent by further processing via SVM for example.

#### 6.2.3. CNN Classifier

**Figure 16:**
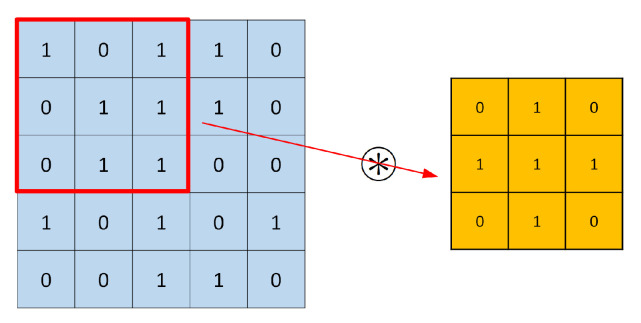
An example of convolutional layer.

**Figure 17:**
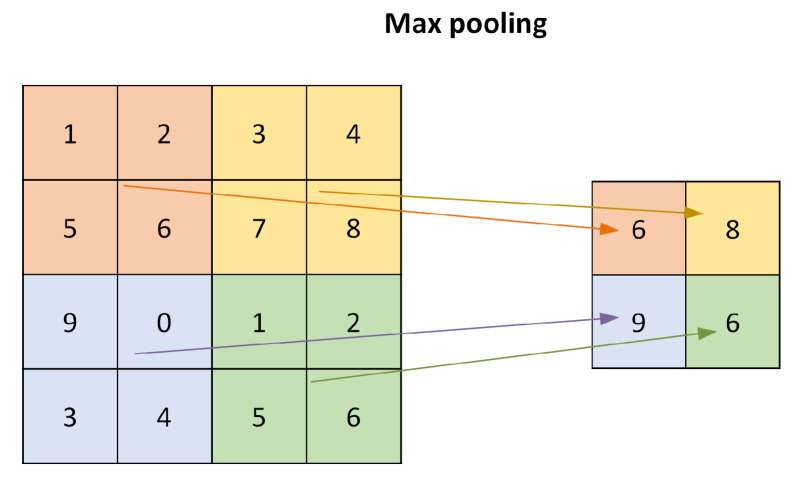
An example of max pooling.

**Figure 18:**
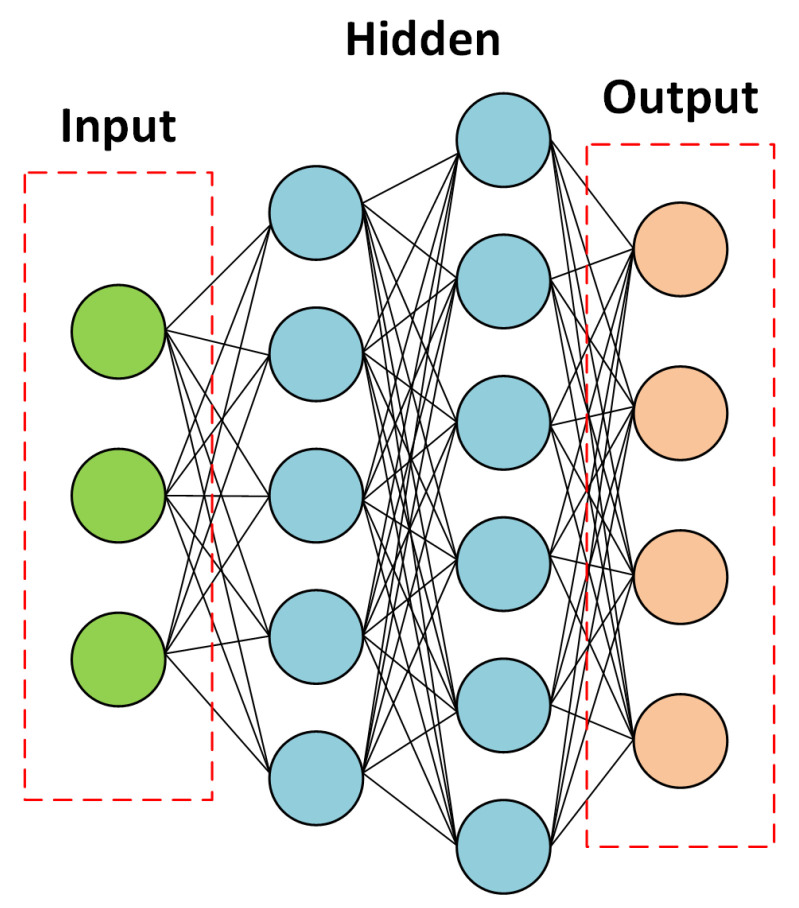
An illustration of a fully connected neural network.

Convolutional neural networks (CNN), the most frequently encountered category of deep learning classifiers, have been widely applied in image identification and pattern recognition. Recently, CNN classifier has also started to be considered as a promising method for the radio identification and RFF [86,87]. A general CNN consists of a combination of convolutional layers, pooling layers, and fully connected layers. This works as the following:

1. The convolutional layer applies a convolution operation between the input signal matrix and a filter (or kernel) (the input signals here are the signals that come to the convolutional layer; the input does not necessarily mean the input data to the beginning of neural networks). For example, Figure 16 considers a 5×5 ‘input’ and a 3×3 filter, the red rectangle selects the same size of data as the filter, then the selected data have a convolution operation with the filter. The red rectangle moves after each convolution operation until all the ‘input’ data experience the convolution operation with the filter.
2. The pooling layer will reduce the number of parameters; it is essentially a sampling method. The common pooling methods are max pooling, average pooling, and sum pooling. Here, we provide an example of max pooling in Figure 17. Max pooling: it chooses the largest number in the selected data.
3. The fully connected layer is the actual neural network, by using the activation function, such as the sigmoid (or logistic function), we are able to label the outputs. A common fully connected layer is made of three parts, the input layer, the hidden layer(s) (also refers to neurons), and the output layer. Figure 18 gives an example of a fully connected neural network. The fully connected neural network can be composed of multiple layers of fully connected neurons. Each layer can be followed by an activation function, such as a relu, sigmoid, or logistic function. The output layer, the last layer of the neural network, commonly uses a sigmoid activation function to assign the probability to each possible class. Figure 18 gives an example of a fully connected neural network.

#### 6.2.4. Other Approaches

Other approaches of ML-based classification less encountered in the context of RFF are: linear discriminant analysis (LDA) [73,82], logistic regression (LR) [88], and random forest [89].

LDA is usually used to separate two or more classes or to achieve dimensionality reduction. The basic idea behind LDA is to find a projection of the input data such that the separation of classes could be maximized. This method is limited, however, by the condition that both input classes follow normal distributions. LR usually works with classes characterized by linear features and it is not well suited to non-linear features as those created by power amplifiers and digital-to-analog converters. The studies in [88], applied in the non-GNSS context, also showed that the SVM outperforms LR. The random forest algorithm is one kind of decision tree used in the classifications, which implements the ‘if-then-else’ logic in order to classify samples. The random forest algorithms are more complex than simple decision-tree algorithms and their complexity is prohibitive complexity for GNSS pre-correlation samples.

## 7. Simulation-Based Example and Feature Down Selection

An in-house-based simulator was built based on Matlab 2020b version and Python 3.7.5. The Matlab modules were used to generate I/Q samples based on a GNSS and a spoofer model, each having five types of transmitter features: PA non-linearities, DAC non-linearities, I/Q imbalance, phase noises, and BPF. The parameters of genuine GNSS transmitters are typically not available in open access, as they are protected via IPR. In the absence of such GNSS exact parameters for these HW features, we adopted various models from the literature. For example, the PA non-linearities were modelled according to [59], and the phase noise existing in the clock unit and up-conversion unit was modelled according to [90]. Details on the parameters used in our simulator are given in Table 2. In order to mimic the characteristics of a sophisticated spoofer, the phase noise of the local oscillator in the spoofer was modelled according to [52], a high-end software-defined radio designed for GNSS signal transmitting and receiving. A simplified model was used for classifying one genuine GNSS transmitter versus one spoofer transmitting GNSS-like signals. As the main goal was to study the feasibility of RFF in the context of GNSS, an ideal, almost noise-free case was considered with a carrier-to-noise ratio (CNR) $C/N_{0}=100$ dBHz. While the noise-free approach is not realistic in real-life scenarios, the purpose here was to show if there is any potential of RFF with pre-correlation GNSS data and to identify which HW features are likely to best differentiate between different transmitters.

A two-millisecond observation window of Galileo E1 band signals was used in the examples shown in this section. In order to deal better with smaller $C/N_{0}$ levels that the ideal case considered here, one could consider the increase in the observation window. However, the simulation times and the complexity of RFF processing would also increase. Under a different randomness seed, we generated 2000 matrices (or images) of genuine GNSS signals and spoofer signals, respectively (thus a total of 4000 inputs to the ML algorithm). Furthermore, the 4000 data inputs were randomly split into 80% of training data and 20% of test data. Such matrices (or images) were the outputs of three considered feature-extraction transforms, namely applied kurtosis, TKEO and spectrogram, applied on the 2 ms observation interval of the raw signal sampled at a very high sampling rate of 491 MHz. Such a high sampling rate was needed in our model because we adopted a quasi-RF model, in order to model the clocks’ non-idealities. The feature-extraction transforms were selected based on the discussions in Section 5, in order to enhance the capability of differentiating genuine GNSS signals from spoofer signals. An SVM classifier, from the scikit-learn library, together with a radial-basis-function kernel was implemented in Python to perform the classification. The grid search method was used to provide the optimized classification results and 100-fold cross-validation on the training dataset were employed to guarantee the convergence of the results.

**Figure 19:**
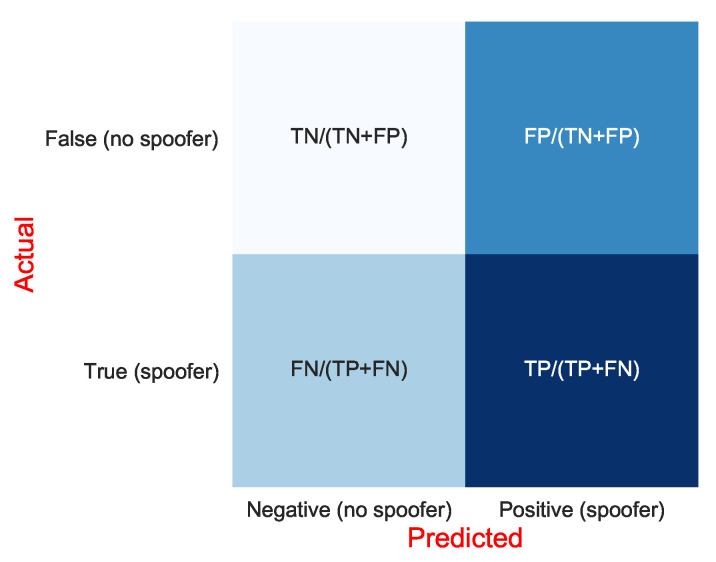
The illustration for normalized confusion matrix. FN is short for false negative rate, FP is short for false positive rate, TN is short for a true negative rate, TP is short for a true positive rate.

The results of the classification are presented via the confusion-matrix metric. Figure 19 illustrates the definition of the confusion matrix used in our work.

**Figure 20:**
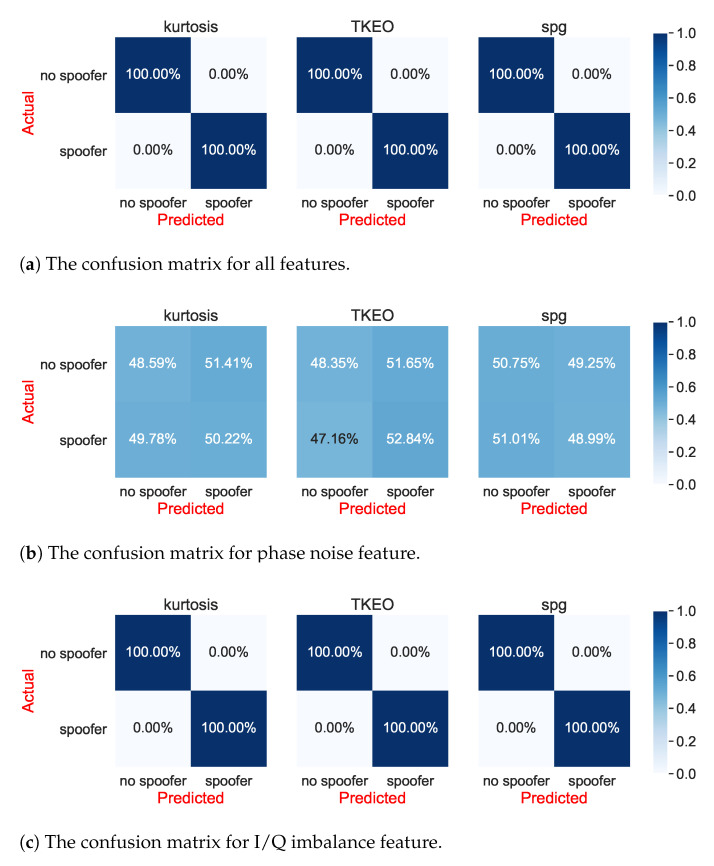

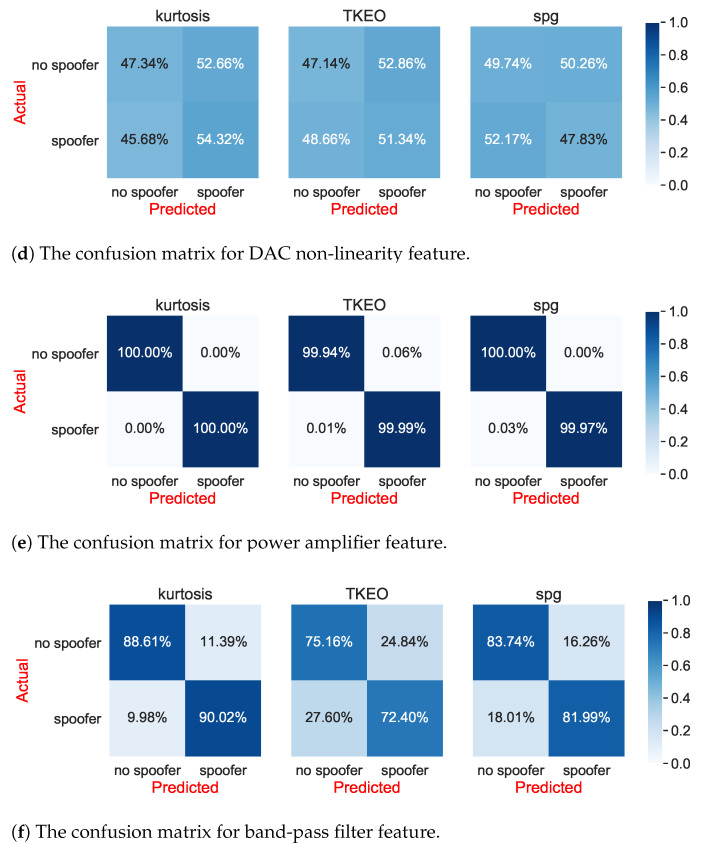
The confusion matrix of a 1 versus 1 scenario under 100 dB-Hz CNR.

In our simulator, each feature can be active or inactive, making the simulator flexible to be able to down select or identify the ‘strongest’ features, as well as their overall impact when they act jointly (as in a realistic transmission scenario). Figure 20 shows the confusion-matrix results, first when all features are combined, and then feature-by-feature, in order to be able to identify which features have a strong impact on RFF and which a have weak or no impact. One very interesting result based on Figure 20 is that, even at a 100 dB–Hz carrier-to-noise ratio, both the phase-noise and DAC-non-linearity features fail to provide differences between the two classes (spoofer present versus genuine Galileo signal present).

Moreover, as seen in Figure 20, the band-pass filter effects can only provide moderate differentiation between the spoofer and GNSS. These results, at a large degree, imply that the phase noise and DAC non-linearity are ‘weak’ features in the GNSS RFF context, while PA and I/Q imbalance, as well as BPF to some extent, are ‘strong’ features. This is also qualitatively illustrated in the next section.

## 8. Comparative Summary of Pre-Correlation RFF Methods in Existing Literature

Table 1 gives a concise survey of main RFF-related studies in the recent literature, by specifying the wireless system under investigation, as well as the main algorithms used for feature detection and classification in those RFF approaches. As seen in Table 1 most of the research work dedicated to RFF has to date been for non-GNSS signals. Moreover, as clearly seen from the last column in Table 1, RFF in the aviation context has been receiving more and more attention in the last two years, e.g., focusing on automatic dependent surveillance-broadcast (ADS-B) surveillance signals and on UAV transmitters and controllers. Table 1 shows that a wide variety of classifiers have to date been investigated in the literature in the context of RFF: from a discrete wavelet transform (DWT) and continuous wavelet transform (CWT) to various neural networks, such as convolutional neural networks (CNN), probabilistic neural networks (PNN) and other machine learning algorithms, such as support vector machines (SVM), subclass discriminant analysis (SDA), multiple discriminant analysis (MDA), or permutation-entropy (PE)-based approaches.

Unlike the typical narrowband terrestrial signals typically studied to date with RFF techniques (see Table 1), the GNSS signals are wideband and continuously transmitted, and hence do not exhibit strong transients to be used as differentiating factors. This means that, for GNSS signals, one should go deeper into the transmitter hardware characteristics and detect the possibly differentiating features between spoofers and genuine GNSS transmitters.

## 9. Qualitative Discussion and Open Challenges

Based on our literature research and the preliminary theoretical analysis, Table 3 shows a suitability analysis of various combinations of feature-extraction transforms and classifiers for four selected classifiers and five selected feature-extraction transforms. The suitability analysis took into account both the expected performance and the complexity of the algorithm.

The most promising combinations, based on our preliminary analysis, are the kurtosis and thresholding combination, and the spectrogram and SVM combination. Potential good results may also be expected, based on a current literature search and theoretical analysis, from kurtosis and SVM combination, as shown in Table 3. Further simulation-based and measurement-based analysis is necessary to validate these findings and this remains a topic of future research. The methodology presented in this paper can serve as a basis for also studying other possible combinations of feature-extraction transforms and classifiers.

Table 4 also discusses the expected impact of various features of the transmitter HW on the accuracy of the results. The analysis is based on the theoretical insights from the mathematical models presented in Section 3. It is expected that the PA non-linearity, the phase noises and the I/Q imbalances are the strongest differentiating features of the transmitter HW impairments, while the DAC non-linearities are expected to have little or no impact upon the classification performance (as differences between the GNSS and spoofer DAC non-linearities are not expected to be high). The band-pass filter (BPF) at the end of the transmission chain is, however, expected to have a negative impact upon the ability to differentiate among various features, because it is acting as a smoother (or high-frequency removing unit). In practice, an RFF algorithm would, most likely, not be able to distinguish between each individual transmitter feature and would treat all effects jointly. Based on sufficiently large databases, it is expected that the positive-impact effects from Table 4 will be predominant compared to the zero- and negative-impact effects.

## 10. Conclusions and Roadmap Ahead

This paper presented a survey of RFF methods for spoofing mitigation in GNSS receivers. While the survey of methods and the methodology presented in here can be generally applied also in a non-GNSS context, the focus in our paper has been on GNSS pre-correlation data, as the pre-correlation anti-spoofing methods are still rare in the current literature.

A four-step methodological approach has been proposed in Section 2, by breaking down the RFF problem into several parts: the effects (or features) occurring at the transmitter side, the channel effects, and the receiver effects. We identified the main sources of possible hardware imperfections (i.e., features) at the transmitter side and we introduced in Section 3 detailed mathematical models for the identified HW impairments for GNSS transmitters. It has also been shown that such HW features are best identified with the help of various feature-extraction time-domain or frequency-domain transforms. Some of the most encountered feature-extraction transforms in the current literature were discussed in Section 5. We also surveyed the literature to identify classification algorithms useful in the context of RFF. Several classification methods, both via thresholding and via machine learning algorithms, were addressed in Section 6. Section 8 provided a qualitative comparison of approaches suitable for GNSS pre-correlation data, based on our literature survey, theoretical modelling, and preliminary simulation-based observations. It is to be emphasized that such RFF algorithms need to be further tested via measurement-based data for understanding their full capacity in a realistic environment, but one of the main take-away points of our research has been that the transmitter HW imperfections do have the possibility to act as differentiating features between spoofers and genuine transmitters if proper combinations of feature-extraction transform and classifiers are found. Our focus has been on the transmitter HW features, but we also discussed the possible effects of the wireless channels and the hardware blocks at the receiver side. To sum up, several challenges remain for the roadmap ahead:

- Addressing the impact of the signal mixtures from signals from various satellites and various frequency bands: typically, the received signal is a mixture of all satellites visible in the sky at the considered moment, and possibly, of one or several spoofing signals. One approach to look at a single signal at a time would be to first despread each signal from each identified pseudo-random code, and then apply successive or parallel interference cancellation methods to identify each signal, one by one. The errors in the estimation of the signals from various satellites would, of course, affect the quality of the re-constructed signal, and possibly, the accuracy of the RFF-based classification. Another approach would be to create huge training databases with all possible mixtures of satellites in the sky and to use those databases in the classification process;
- Evaluating and mitigating the impact of channel multipath and fading effects: each wireless channel (from satellite or spoofer) has its own random signature, determined by the multipath delays, Doppler spreads, and fading effects. As these effects are random in nature, they will, most likely, not provide additional ‘features’, but will have a negative impact on the strength of the transmitter features. The effect of the wireless channels upon the RFF algorithms can be further investigated via simulation- or measurement-based approaches and it remains a topic of future investigation;
- Understanding the impact of the receiver HW features upon the RFF methods: while the same receiver is capturing either genuine GNSS signals or a mixture of genuine signals and spoofer(s), and thus the same receiver effects are present in both situations (spoofer present or spoofer absent), the receiver also has local oscillators, ADC and filter blocks, etc., and each of them can introduce additional phase noises, non-linearities and I/Q imbalances. Intuitively, such effects will have a negative impact upon the classification accuracy compared to an ideal receiver (without any HW imperfections), but such effects need to be further analysed based on measurements or simulated data.
- Dealing with the negative impact of high noise levels on RFF performance, especially when dealing with low-power signals such as those in the pre-correlation domain: GNSS signals in urban scenarios, such as GNSS receivers on-board of drones flying through tall buildings, can be received at relatively low CNRs, and these low CNRs are likely to act as smoothers of the transmitter features, to the point of fading them out. It remains an open research question what the CNR threshold is above which the RFF methods with pre-correlation GNSS samples are likely to work;
- Validating through real-field measurements the promising RFF performance for authenticating GNSS signals.

One of the main contributions of our paper was presenting a step-by-step methodological approach proposed to be adopted for a designer wishing to build an RFF algorithm in a GNSS receiver. The identified transmitter HW features are likely to be reflected not only in the pre-correlation data (illustrated in our examples through the paper), but also in the post-correlation and navigation domains, thus our four-step methodology also paves the road towards more advanced RFF GNSS processing in all three domains (pre-correlation, post-correlation, and navigation), with a future aim to offer robust and hybrid anti-spoofing solutions. An additional contribution of this paper has been to present an ample survey of existing RFF methods in the literature used with both GNSS and non-GNSS signals and already showing promising results. As described in this last section, several challenges are still to be overcome towards the success of RFF methods, especially when relying on the low-power GNSS I/Q raw data. It is our belief that this survey bridges the missing gap between the RFF studies in the non-GNSS context and the anti-spoofing methods studied to date only at the post-correlation and navigation levels in the GNSS context. It is our intent that this paper sheds new light on how to approach an RF fingerprinting process to identify hidden transmitter features, by first decomposing the problem into the relevant transmitter features and then by selecting the most suitable pair of feature-extraction transform and classifier algorithm in order to classify the transmitters according to their features or HW impairments. While many challenges still remain in the RFF GNSS research field, it is also the authors’ belief, based on our understanding of the research problem, that by combining various authentication methods, at different levels (pre-correlation, post-correlation, and navigation levels), one is more likely to obtain good results than by using a single authentication method. The simulation-based results presented here are only for some selected illustrative parameters and are useful in the context of down selecting the most important HW features of a GNSS transmitter. We saw that, even under ideal conditions such as 100 dBHz carrier-to-noise ratio, the phase noise and the DAC nonlinearities are not differentiating features, while P non-linearities, I/Q imbalances, and band-pass filters carry the potential of being good RF ‘fingerprints’. For the sake of a reduced complexity of simulations, the observation window used in our simulations was of 2 ms. Further investigative studies at a lower $C/N_{0}$ than 100 dBHz should also increase the observation windows, in order to deal better with the high noise level typical in the pre-correlation domains. The equivalent block diagrams and the methodological approach presented here, as well as the initial pre-selection of relevant features and feature extractors can also serve the basis towards further studies in the post-correlation domain, where the noise levels are significantly lower than in the pre-correlation domain, especially for the long post-detection integration times.

## Figures and Tables

**Table 1:** Overview of state-of-the-art: RFF-related studies based on pre-correlation data, for wireless communications, and navigation applications.

Ref., Year	Studied Signal Types	Studied Algorithms	Detection Performance Metrics Given?	Using I/Q (or Pre-Correlation Data)?	Domain
[62], 2003	Bluetooth and WiFi	Bayesian step detector of transients	No	Yes	IoT
[63], 2006	Ethernet devices	Matched filtering	No	No	Cable networks
[60], 2007	Chipcon sensors at 433 MHz carrier	DWT	No	Yes	IoT
[64], 2008	WiFi	Support vector machines (SVM) and CNN	No	Yes	IoT
[65], 2009	QPSK and DQPSK modulated narrowband signals	Maximum likelihood classification	No	Yes	IoT
[22], 2010	WiFi and 4G/LTE	Analysis of variance (ANOVA) classification	No	Yes	Cellular
[66], 2012	TDMA satellites with QPSK modulation	SDA	No	Yes	Satcomm
[48], 2014	16-APSK modulated narrowband signal	Analytical study	No	No	IoT
[67], 2015	UWB noise radar	MDA	Yes	No	Radar
[68], 2003	**GNSS**	Allan deviation and time interval error	Yes	No	GNSS
[24], 2017	nRF24LU1+ IoT devices at 2.4 GHz	Permutation entropy (PE) and dispersion entropy (DE) with SVM	Yes	Yes	IoT
[69], 2017	GMSK-modulated narrowband signals	Normalized PE	No	Yes	IoT
[21], 2017	**GNSS**	Allan deviation and time interval error	Yes	No	GNSS
[56], 2017	WiFi	Probabilistic neural network (PNN) classifier	No	Yes	IoT
[70], 2018	**GNSS**	Polarization vector with dual antennas	No	No	GNSS
[71,72], 2019	Cellular signals	Kurtosis	No	Yes	Cellular
[25], 2019	GSM	Continuous wavelet transform (CWT) and CNN	Yes	Yes	Cellular
[73], 2019	IoT amplifiers	Linear discriminant analysis (LDA)	Yes	Yes	IoT
[74], 2019	AM-modulated signal	CNN	Yes	Yes	IoT
[75], 2019	QPSK-modulated narrowband signals	Hilbert–Huang Transform (HHT) and CNN	Yes	Yes	IoT
[76,77], 2020	ADS-B signals	CNN	Yes	Yes	Aviation (surveillance)
[78], 2020	UAV controller	SVM, random forest, neural networks	Yes	Yes	Aviation (UAVs)
[79], 2020	ADS-B signals	CNN, message structure aided attentional convolution network (MSACN)	Yes	Yes	Aviation (surveillance)
[80], 2020	Wimax transmitters	SVM	Yes	Yes	IoT
[81], 2020	UAV transmitters	Neural networks	Yes	Yes	Aviation (UAVs)
[82], 2021	ZigBee signals	Gaussian probabilistic LDA	Yes	Yes	IoT

**Table 2:** Parameters in simulation.

Parameters	Value	
Observation interval (ms)	2	
Galileo band	E1	
Intermediate frequency (MHz)	61.38	
Maximum Doppler shift (kHz)	5	
TX filter bandwidth (MHz)	100	
**Parameters Used in Genuine GNSS Simulator**		
DAC phase noise	Frequency offset (Hz)	Level (dBc/Hz)
	1	−90
DAC non-linearity	$y=x-{0.0038x|x|}^{2}$	
Clock unit phase noise	Frequency offset (Hz)	Level (dBc/Hz)
	1	−95
	10	−125
	100	−135
Clock unit non-linearity	Ignored	
Up-conversion unit phase noise	Frequency offset (Hz)	Level (dBc/Hz)
	1	−50
	10	−70
	100	−95
Up-conversion unit I/Q imbalance	Amplitude (dB)	Degree
	1	3
Band-pass filter	See Figure 6a	
**Parameters Used in Spoofer Simulator**		
DAC phase noise	Frequency offset (Hz)	Level (dBc/Hz)
	10	−50
	100	−70
	500	−85
DAC non-linearity	$y=x-{0.05x|x|}^{2}$	
LO phase noise	Frequency offset (Hz)	Level (dBc/Hz)
	1	−80
	10	−110
	100	−135
Mixer I/Q imbalance	Amplitude (dB)	Degree
	3	5
Band-pass filter	See Figure 6b	

**Table 3:** Preliminary analysis on the suitability of various feature-extraction transforms for various classification methods (+ = low, ++ = medium, +++ = high) in the context of pre-correlation GNSS data.

Classifier Type	Feature Extraction Transform					
	EVM	Kurtosis	TKEO	Spectrogram	CWT	DWT
**Classification via kNN**	+	+	+	+	+	+
**Classification via SVM**	+	++	+	+++	+	++
**Classification via CNN**	+	+	+	+++	+	+
**Classification via Thresholding**	+	+++	+	+	+	+

**Table 4:** Preliminary analysis on the impact of various hardware features upon the capacity to distinguish between transmitters, based on Section 7: 0 = no impact, + = positive impact (i.e., can increase the RFF accuracy).

	Transmitter Features				
	Phase Noise	I/Q Imbalance	DAC Non-Linearity	PA Non-Linearity	BPF
**Impact**	0	+	0	+	+

## Data Availability

Not applicable.

## Abbreviations

The following abbreviations are used in this manuscript:

ADC	Analog-to-Digital Converter
AGC	Automatic Gain Control
ANOVA	Analysis of Variance
APSK	Amplitude and Phase Shift Keying (modulation)
BLE	Bluetooth Low Energy
BPF	Band-Pass filter
BPSK	Binary Phase Shift Keying (modulation)
CDMA	Code Division Multiple Access
CNR	Carrier-to-Noise Ratio
CNN	Convolutional Neural Networks
CWT	Continuous Wavelet Transform
DAC	Digital-to-Analog Converter
DE	Dispersion Entropy
DQPSK	Differential Quadrature Phase Shift Keying (modulation)
DWT	Discrete Wavelet Transform
ESA	European Space Agency
ESTEC	European Space Research and Technology Centre
GNSS	Global Navigation Satellite System
GPS	Global Positioning System
GLRT	Gaussian Likelihood Ratio Test
FE	Front-End
FIR	Finite Impulse Response
GNSS	Global Navigation Satellite System
GSM	Global System for Mobile Communications
HHT	Hilbert–Huang Transform
HW	Hardware
IoT	Internet of Things
I/Q	In-Phase /Quadrature
LDA	Linear Discriminat Analysis
LPA	Low Power Amplifier
LO	Local Oscilator
LRT	Likelihood Ratio Test
LTE	Long-Term Evoloution
MDA	Multiple Discriminant Analysis
MSACN	Message Structure Aided Attentional Convolution Network
OXCO	Oven Controlled Crystal Oscillator
PA	Power Amplifier
PCA	Principal Component Analysis
PE	Permutation Entropy
PN	Phase Noise

PNN	Probabilistic Neural Networks
PSD	Power Spectral Density
QPSK	Quadrature Phase Shift Keying (modulation)
RF	Radio Frequency
RFF	Radio Frequency Fingerprinting
SDR	Software Defined Radio
SNR	Signal-to-Noise Ratio
SVM	Support Vector Machines
SW	Software
TCXO	Temperature Controlled Crystal Oscillator
TDMA	Time Division Multiple Access
TKEO	Teager–Kaiser Energy Operator
UAV	Unmanned Aerial Vehicles
USRP	Universal Software Radio Peripheral
UWB	Ultra Wide-Band
